# Mothers’ involvement in providing care for their hospitalised sick newborns in Kenya: a focused ethnographic account

**DOI:** 10.1186/s12884-023-05686-3

**Published:** 2023-05-26

**Authors:** Dorothy Oluoch, Lisa Hinton, Mike English, Grace Irimu, Truphena Onyango, Caroline O. H. Jones

**Affiliations:** 1 Centre for Geographic Medicine (Coast), KEMRI-Wellcome Trust research programme, Nairobi, Kenya; 2 THIS Institute, University of Cambridge, Cambridge, UK; 3 Centre for Tropical Medicine and Global Health, NUFFIELD department of medicine, University of Oxford, Oxford, UK; 4 Department of Paediatrics and Child Health, University of Nairobi, Nairobi, Kenya

**Keywords:** Neonatal, Mother's roles, Participation in care, The context of newborn care, Newborn unit, Family-centred care

## Abstract

**Introduction:**

There is growing evidence that parental participation in the care of small and sick newborns benefits both babies and parents. While studies have investigated the roles that mothers play in newborn units in high income contexts (HIC), there is little exploration of how contextual factors interplay to influence the ways in which mothers participate in the care of their small and sick newborn babies in very resource constrained settings such as those found in many countries in sub-Saharan Africa.

**Methods:**

Ethnographic methods (observations, informal conversations and formal interviews) were used to collect data during 627 h of fieldwork between March 2017 and August 2018 in the neonatal units of one government and one faith-based hospital in Kenya. Data were analysed using a modified grounded theory approach.

**Results:**

There were marked differences between the hospitals in the participation by mothers in the care of their sick newborn babies. The timing and types of caring task that the mothers undertook were shaped by the structural, economic and social context of the hospitals. In the resource constrained government funded hospital, the immediate informal and unplanned delegation of care to mothers was routine. In the faith-based hospital mothers were initially separated from their babies and introduced to bathing and diaper change tasks slowly under the close supervision of nurses. In both hospitals appropriate breast-feeding support was lacking, and the needs of the mothers were largely ignored.

**Conclusion:**

In highly resource constrained hospitals with low nurse to baby ratios, mothers are required to provide primary and some specialised care to their sick newborns with little information or support on how undertake the necessary tasks. In better resourced hospital settings, most caring tasks are initially performed by nurses leaving mothers feeling powerless and worried about their capacity to care for their babies after discharge. Interventions need to focus on how to better equip hospitals and nurses to support mothers in caring for their sick newborns, promoting family centred care.

**Supplementary Information:**

The online version contains supplementary material available at 10.1186/s12884-023-05686-3.

## Introduction

Reducing newborn mortality remains a global priority [1]. The majority of the 2.76 million deaths that occur among neonates each year happen in low and middle-income countries (LMICs) and progress in reducing neonatal mortality in these settings has been slow [2, 3]. Although Kenya is successfully reducing child mortality, little progress has so far been made in reducing deaths among newborns (0–28 days old). Improving the quality and access to care for sick newborns is central to reducing this burden [4, 5]. While ensuring effective clinical management is vital to the survival of sick newborns, there is a growing body of evidence suggesting that social factors and caregiving processes are also important in shaping neonatal health outcomes [6, 7]. The recently published standards for improving the quality of newborn care in health facilities published by the World Health Organisation (WHO) [8] include new quality statements regarding the need to ensure that all carers receive counselling and education about the baby’s illness (standard 4.5), that families are recognised as partners in care (standard 6.3) and that all small and sick newborns have access to a sufficient health care staff at all times according to standard levels of care (standard 7.1).

Involving parents in the care of hospitalized sick newborns can help in avoiding poor outcomes and reduce stress among parents [9–11]. There is also increasing evidence that maternally delivered interventions such as emollient therapy [12], kangaroo mother care [13–15] and preterm infant massage [16] have positive effects on the survival and long term development of low birth weight and premature neonates [12–15]. Parental involvement in the care of their sick newborn has been highlighted in several studies from high income countries (HICs) as an important element of the effective implementation of family centred care (FCC) [17–20]. These studies also note that involvement in care results in better mother-infant bonding and attachment and, as such, improved parental experiences. Subsequently, with increased involvement, there are better rates of breastfeeding, kangaroo mother care (KMC), and ultimately shorter hospital stays [21, 22]. Most recently, a multi-country randomised control trial implemented by the WHO in Ghana, India, Malawi, Nigeria and Tanzania found that immediate KMC (iKMC) for sick newborns with mothers present with their babies in the hospital newborn unit (NBU) 24 h per day aiming for 20 h a day of KMC from within 2 h of birth resulted in a 25% reduction in mortality in the first 28 days of life in the iKMC arm compared with standard KMC [23]. In addition to the potential for enhancing newborn survival, involving parents in newborn care has the potential to enhance parenting skills and to improve their perceptions of the care their child receives. Perceptions of the appropriateness of care is central to encouraging uptake of services, increasing compliance, and potentially enhancing recovery and later development outcomes [24, 25].

Currently however, the formal norms of care provision in new born units (NBUs) in most settings are that the tasks involved in caring for hospitalised sick newborns are handed over to nurses and other health professionals [26, 27]. While nurses are central to the provision of care in NBUs, many low- and middle-income countries (LMICs) struggle to meet the ratios of staff to babies recommended by national standards bodies [28, 29]. In the UK, the minimum standards for nurse staffing levels for each category of neonatal care are 1:1 for neonatal intensive care, 1:2 for neonatal high dependency care and 1:4 for neonatal special care [30]. Similar standards are found in the European standards of care for newborn health, with a recommended ratio of 1:1 for the very sick/critical babies and a ratio of 1:2 for relatively stable babies [31]. India recommends 1:3 or 1:4 for special care; and South Africa recommends 1:1 or 1:2 for intensive care, 1:2 or 1:3 for high-dependency care and 1:6 for standard inpatient or kangaroo mother care units [8]. These ratios are in stark contrast to the ratios of witnessed in many LMICs and a recent analysis of factors influencing the delivery of quality newborn care in 12 countries in Africa and Asia found that shortages of neonatal nurses was a key problem [32].

Kenya has no published standards for staff to baby ratios. A recent study of neonatal nursing conducted across 33 hospitals in Nairobi found a low nurse to baby ratios of 7–15 babies per nurse [29]. Studies have documented the effects of these high baby to nurse ratios both on the ability of nurses to provide care and the types of care that are prioritised or missed with the NBU [23, 33–35]. There is a growing body of evidence that as a way of coping with the high workloads exacerbated by understaffing, nurses prioritise clinical tasks over other tasks within the newborn units [36]. This raises the question as to what happens to the missed clinical care? For example, who undertakes the primary tasks not completed by the nurses, such as washing the baby, changing the diapers, and more specialised tasks such as overseeing the placement of eye masks for babies in phototherapy and even nasogastric tube feeding? Studies in NBUs in Iran where the nurse to baby ratio ranged from 1:5 to 1:8 found that unplanned and informal delegation of care by the nurses to the parents was common with little or no parental training [33]. These studies also found that the parents’ needs were neglected by the clinical staff with support being provided by the informal parent to parent support networks that developed in the NBUs.

Studies that examine how mothers are involved in providing care for their hospitalised newborns, and could address these gaps in care, are scarce. To the best of our knowledge, there is no systematically collected and published information on who provides the day-to-day non-clinical or ‘primary’ caring roles for the particularly vulnerable babies in neonatal units in the highly resource limited settings found in many NBUs in sub-Saharan Africa where the baby to nurse ratio far exceeds international recommendations. Understanding the tasks that mothers undertake and the support they receive to carry out these tasks is central to identifying interventions to improve sick newborn care and enhance staff and the families’ ability to partner in delivering quality care. The focus of this paper is to: (i) provide insight into the roles that mothers play in caring for their sick newborns in two contrasting NBUs in Nairobi, Kenya; (ii) to critically examine how the structural, economic and social context shape the nature of mothers’ participation in care; (iii) identify key gaps and discuss opportunities for improving NBU care in line with the WHO standards in such highly resource constrained settings.

## Methods

### Study design and setting

Data for this paper are drawn from a qualitative study that employed an ethnographic approach [37, 38] to examine the context of care inside the NBU in one public and one faith-based hospital in Nairobi, Kenya.

### Participants and sampling

The participants were mothers of hospitalised small and sick newborns. The two hospitals were purposively selected based on the provision of inpatient neonatal care 24 h 7 days a week, their patient volumes, ease of access, and willingness to allow observers on the ward for extended periods. Mothers taking part in the discharge interviews were purposively selected.

### Data collection

Data were collected through non-participant observations in the NBUs of the two hospitals and in-depth interviews with purposively selected mothers at the point of the discharge of their baby from the hospital. The focus of the observations was the roles that mothers played in caring for their baby born in the hospital and immediately admitted to the newborn unit. The approach to data collection, analysis, and interpretation was informed by critical medical anthropology [39, 40]. Through observations, our attention increasingly focused on examining the context (structural, economic, and social) of care provision and the social relationships that shaped newborn care in the two hospitals. The discharge interviews were guided by a semi-structured interview guide developed through the observations and designed to follow up on issues that arose during the non-participant observations and informal conversations. The interviews were conducted either in English or Swahili depending on the choice of the participant. The data were collected between March 2017 and August 2018.

During the study, the first author (DO) and her research assistant (TO) spent 4 months in each hospital conducting approximately 627 h of non-participant observations [41]. Observations covered day and night shifts and were spread over weekdays and weekends. Alongside the observations, informal conversations with the mothers present in the newborn units were a critical source of information in this study. These day-to-day conversations were unstructured and enabled follow-up on issues that were observed while in the NBU [42]. The non-participant observations focused on the context of care, tasks, and roles that the mothers played, and the nature of the relationships that existed within the ward.

A total of 40 discharge interviews were undertaken (20 in each hospital) with purposively selected mothers. Selection of the mothers was based on the condition of the baby at discharge and the willingness of the mother to participate in a formal interview. The demographic details of these interview participants are provided in Annex 1.

Data were in the form of field notes which were kept daily and reviewed weekly by DO and TO.

### Data analysis

A modified grounded theory approach [43] was used in the analysis of these data with DO, LH, and CJ meeting monthly to review emerging themes and plan for subsequent data collection. Additionally, in the analysis and interpretation of the data, we adopted a Critical Medical Anthropology (CMA) lens, an approach to understanding how health inequities are shaped by social and economic structures and institutions that create, enforce and perpetuate observable disparities in health [44]. The approach emphasizes “the importance of political and economic forces, including the exercise of power, in shaping health, disease, illness experience, and health care” [40].

## Results

The results describe the contrasting economic and structural contexts of the two NBUs, variations in the participation by mothers in the care of their baby and the views of the mothers on their participation in their baby’s care.

### The economic context of newborn care

The two hospitals, located in different neighbourhoods in Nairobi, served very different populations. The faith-based hospital was in an affluent neighbourhood while the government funded hospital was located next to a slum. Most of the women seeking care from the faith-based hospital were in full-time employment and had attained post-secondary level schooling. Many of these women had access to medical health insurance which catered for hospital admission costs for themselves and their babies. At admission to the faith-based hospital NBU, a mandatory deposit of approximately USD 300 was required. In addition to this mandatory deposit, the daily cost of admission for a bed for the mother, and a cot and treatment for the baby ranged from a minimum of USD 35 to a maximum of USD 150. There was also the daily doctor’s fee which ranged from USD 20 to 35 per day, depending on the doctor treating the baby. The average monthly minimum wage for Nairobi is approximately 188 USD (KNBS, Economic survey 2021).

By contrast, many of the mothers in the government funded hospital were either unemployed or worked in the informal job sector and lacked any form of medical insurance cover. The care in the government funded hospital was supposed to be free, but most mothers incurred some costs for necessary medication and tests which were not available in the hospital. In the public hospital, mothers were expected to stay throughout their baby’s admission. Mothers in the faith-based hospital had the option of early voluntary discharge and due to the daily bed costs, many mothers chose this option, leaving behind their babies under the care of the nurses.

### Nurses to baby ratios and technologies of care

The essential biomedical technologies for neonatal care were available in both NBUs. Both had functioning incubators, radiant warmers, phototherapy machines, and other essential basic equipment. A recent study of the availability of equipment supporting care across NBUs in Nairobi found that public hospitals were better equipped than faith-based hospitals, but due to patient pressure, much of the equipment in government funded hospitals had to be shared [29]. This was mirrored in our observations with babies in the government funded hospital often sharing incubators and phototherapy machines, a practice never observed in the faith-based hospital.

There were also stark differences in nurse to baby ratios. In the government funded hospital, we observed that often one nurse could be caring for 15–40 newborns during any given shift. This compares to our observation in the faith-based hospital of a nurse to newborn ratio of 1:1 for the Neonatal Intensive Care Unit (NICU) and a maximum of 1:4 for stable babies.

Despite the observations of inadequate nursing cover in the government funded hospitals, the mothers themselves did not appear to be particularly concerned about a lack of staff during the day. This might be because during the day there were often several professional staff in the ward including nutritionists, intern clinical officers, and nursing students who were present attending to the babies and the mothers. However, the mothers were very concerned about the lack of staff at night (6:00 pm to 6:00 am). As highlighted by one participant from the government funded hospital:

*“I feel that quality of care in the facility was good, especially during the day, because they are always there, however at night when there is only one nurse, it is just you and your God”.***(DI_K004)**.

### Diverse environments of care

#### Chaos versus calm

Entering the NBUs of each hospital was like stepping in to two different worlds. The entrance to the maternity ward and the NBU in the government funded hospital was crowded, noisy, and to an outside observer, appeared chaotic. In the corridors and public waiting areas, the sights and sounds of childbirth were clearly visible and audible. The NBU was located on the first floor, above the maternity ward, and women in labour were often found sitting or pacing around in the corridor and stairs leading up to the NBU. The air had a slightly fetid, unpleasant odour; a combination of blood, body odour, and the damp mops used in cleaning the floors. While access to the NBU was controlled (through a usually guarded grilled door) and visitors were restricted, during the day it was always very busy with the mothers sitting by their baby’s incubator or cot and nurses, clinical officers and nursing students on attachment carrying out their work. The postnatal ward where mothers were supposed to stay while their baby was an in-patient was on a different floor and some way from the NBU and often there were insufficient beds for all the mothers. Consequently, mothers often spent their time in the NBU on chairs next to their baby. The space was stuffy, hot, and filled with the beeping sounds of machines. The smell inside was not much better than outside the NBU and the floor was mopped only once a day. Outside the NBU there was a waiting area with two wooden benches where relatives would sit and wait for the mothers to come out of the ward, or on occasion, be let into the ward to visit a mother and baby. This bench was constantly full with relatives waiting to visit the mothers.

By contrast, the single waiting area for maternity and the NBU in the faith-based hospital was calm, quiet, organised, clean and fresh smelling. Mothers in labour were kept inside the maternity ward and there were few signs or sounds of the pains of childbirth. The joint waiting area outside the maternity ward and NBU of this hospital was rarely occupied as the visitors and family members could sit in the private rooms where the mothers slept. These rooms were along the corridor opposite the NBU. Inside the NBU, there was an air of quiet organised calm; the floor was covered in white tiles; the care spaces were separated by glass partitions covered with pinkish light curtains that were decorated with a warm coloured cartoon print. The floors were mopped thrice a day and the air smelt fresh and clean. Mothers were not permitted to be continuously present in the NBU and during allotted visits to their baby they would usually sit in the breastfeeding room, tucked away from the ward. Here, there were no students on attachment or clinical officers, just the nurses caring for the babies.

### Participation by mothers in care

**Fig. 1:**
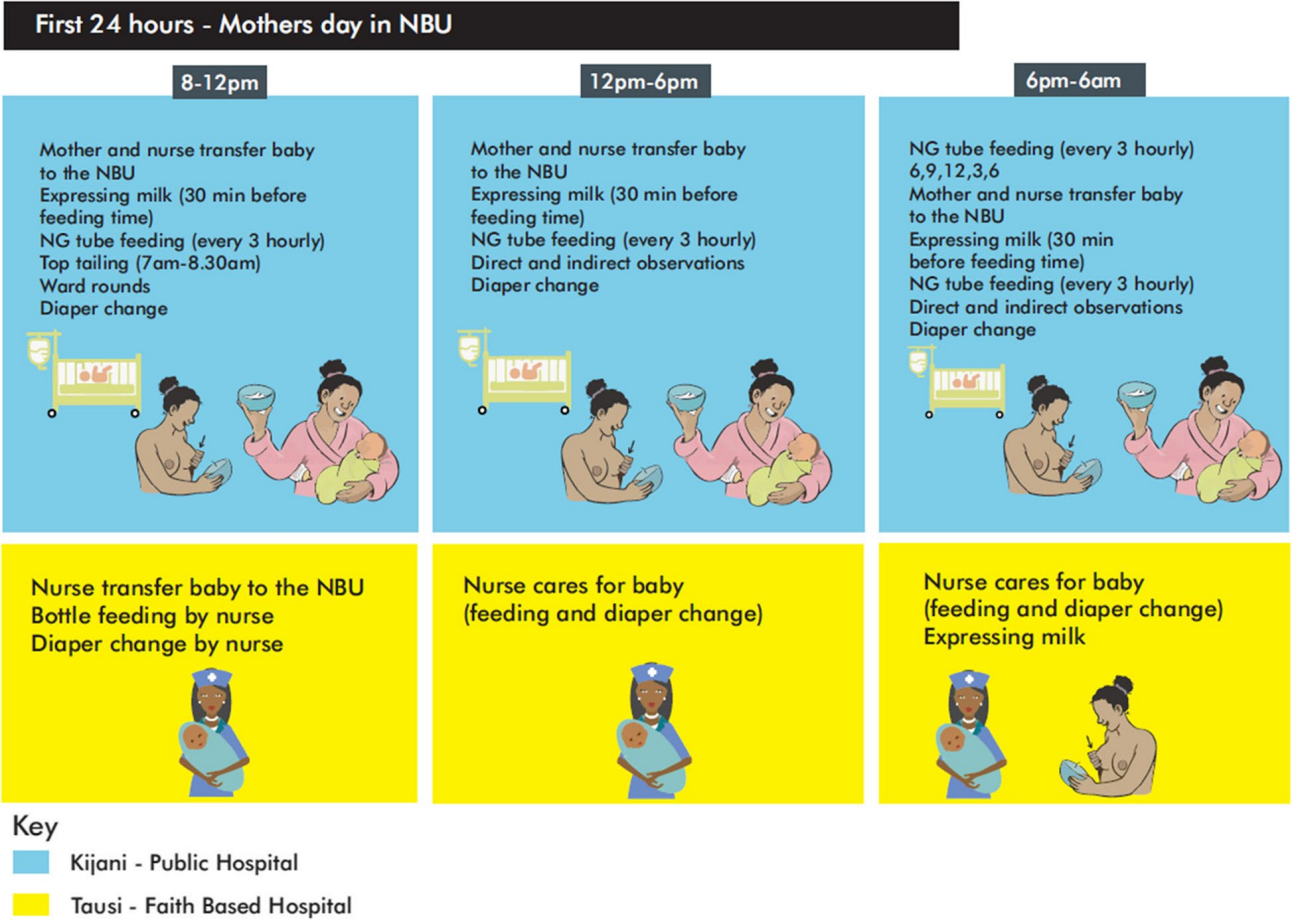
First 24 hours

To an observer, the roles that a mother undertook, and her involvement in the care of her baby, were very different in each NBU. In the government funded hospital, mothers who had experienced a trouble-free delivery and were able to walk up to the NBU, on their own or accompanied by a nurse, immediately became involved in providing care for their babies. As is described in Fig. 1; Table 1, within the first 24 h of their baby’s admission, mothers, even those with babies that needed to be placed in a resuscitator, became involved in providing some level of care. The main task performed by the mothers during the first 24 h of admission in the government funded hospital was diaper changing, top tailing (washing the baby) and, for those who were not on the resuscitator, feeding. A few mothers with full-term babies with jaundice or an infection were able to breastfeed their baby. Mothers of premature babies who were not on oxygen therapy expressed milk and fed their babies through a nasogastric (NG) tube once that had been inserted by a nurse. The following description of the NG tube feeding from our observations provides an illustration of how the mothers in the government funded hospital learnt how to provide care for their babies and from where they received support.

**Table 1:** Summary of mother’s daily activities

		Comparison	
**PHASE**	**TASKS**	**Government funded hospital**	**Faith-based hospital**
**Critical Phase**	• *Feeding* • *Top tailing/ Bathing* • *Diaper change* • *Observations*	Mother takes on caring tasks within 24 h of admission.	Mother takes on caring tasks > 24 h after admission.
		Mother NG tube feed baby.	Nurse NG tube feeds or bottle-feeds baby.
		Mother top tails baby.	Nurse bathes baby.
		Mothers present throughout the NBU and directly observe their babies. (Alerting nurses, fixing oxygen masks out of place, turning babies, fixing eye protection during phototherapy).	Mothers are absent from the NBU and therefore no direct observation of their baby.
**Stable phase**	• *Feeding* • *Top tailing/ Bathing* • *Diaper change* • *Observations* • *KMC* • *Giving oral medication*	Mothers give oral medication.	Mothers do not give oral medication.
		Mothers breastfeed and some continue to NG tube feed.	Mothers breastfeed.
		Mothers top tail (sponge bathe).	Mothers bathe their babies.
		Mothers place baby on phototherapy after breastfeeding.	Mothers place baby on phototherapy after breastfeeding.
		Mothers change diapers.	Mothers change diapers.
		Mother practices continuous KMC and mothers continue to observe their own baby in the KMC room.	Mother practices intermittent KMC.

#### NG tube feeding

After the initial fixing of the NG tube by a nurse, the mother would be given syringes for measuring the milk that she was expected to express and given brief instructions by a nurse or a nutrition intern on how to administer the milk through the tube.  The following is an illustrative explanation given to one of the mothers by a nutritionist intern on how to feed her baby:

*Get a cup, express some milk then measure with this syringe. Connect this syringe to this tube and transfer the measured milk. When done remove the syringe and cover the pipe like this…*

During such explanations the mothers would rarely ask any questions. In some instances, the staff fixed the NG tubes but offered no explanation about how to feed the baby. In such cases the mother would ask a mother sitting next to them for help or they would turn to a nurse. In many instances the nurses would ask the mother to ask their neighbours, referring to other mothers, for help. To provide an example, Table 2 contains a description of a mother’s experience with NG tube feeding.

**Table 2:** Description of NG tube feeding of a premature baby, First 24hrs in Kijani – room A

Among the three other mothers whom nurse Mwajuma had shown where to place their babies was Hellen. Hellen is a first time mother and she was told to place her baby in an incubator that already contained another premature baby. For the first one hour, she mostly just stares at what is happening around her. Next to her are other mothers including the mother of the baby that is in the incubator with her baby, all of whom are busy either expressing milk or feeding. Nurse Mwajuma is still attending to the baby in the radiant warmer and Hellen appears to have no idea what is expected of her or what she is to do. She keenly observes what the other mothers are doing and strikes up a conversation with the mother of the baby who shares the incubator with her baby. Her neighbour had been there for a couple of weeks. Under each incubator are drawers and her neighbour informs her that she can keep her babies diapers and feeding cups in the drawer. After checking, Hellen realises that her baby needs a diaper change and goes down to the postnatal ward to bring some diapers, she had brought with her essential baby items such as clothes and diapers which she had left in the postnatal ward. Upon her return, she turns to one of the mothers next to her and asks how it is done, she asks how to open the incubator and how to change the diaper. Having been there for some days now, her neighbour knows how to open and close the incubator and Hellen observes and learns from her neighbour. She is instructed by the mothers next to her on how to go about diaper change and how to close the incubator when done.			
Mwajuma passes by and tells her to express milk and feed the baby. *“She doesn’t have this pipe”,* she informs Mwajuma who then tells her and asks her to wait a bit. As nurse Mwajuma goes away, Hellen turns to the mother next to her and asks, “*Where do I get the cup and how do I know how much I am to feed?”* she asks her neighbour. *“The nurse will fix for you this pipe and tell you how much to feed”,* she is told. She sits and waits for Mwajuma to get to her baby. When the nurse gets to her, she stands behind the nurse wanting to observe what the nurse was doing. However, immediately she notices the NG tube being inserted in her baby’s nose, she moves back and looks the other way, with both hands on her cheek. She can’t bring herself to observe the procedure. One of the mothers next to her smiles and asks her what was wrong to which she responds that she can’t watch that pipe being fixed, she imagines that the baby is feeling pain.			
After fixing the NG tube, the nurse provides her with feeding syringes and tells her *“do you have a cup, get a cup and express milk, then measure 5 mls and then feed the baby, I will get you the syringe, so you measure and then pour into this pipe”.* She is handed the syringes which she places on the drawer on her baby’s incubator and proceeds to get a cup. She is advised by the mother she is sharing the incubator with to go and buy a cup downstairs by the gate. Hellen leaves the NBU and after a few minutes she walks back in with a cup, sits back on the chair and begins expressing. Having seen what the other mothers were doing, she follows suit and does what she saw the other mothers do. Unsure of what to do next after expressing, she turns to her neighbour and asks how to measure. *“Put what you have expressed into this syringe, you see this lines and numbers here, pointing to the marks on the syringe, so this is one..two,…five, so you put until here and then you open here and pour the milk inside the pipe”.* After feeding her baby she is shown where to wash her cup and she then keeps her feeding cup and syringes on her drawer.			

Over the course of the observation period, there were days when, during NG tube feeding, a baby would choke and vomit which caused panic with the concerned mother screaming for help from the nurses. Mothers had developed two main ways which they adopted to prevent this choking, one practical, and the other very much influenced by cultural beliefs. The practical trick some mothers used involved devising a way of controlling the flow of milk in the tube. They would pour some milk in the syringe and then pinch, fold and hold the pipe. Many mothers were seen to be doing this and upon asking they told DO that they did this to regulate the flow of milk. If they felt the milk was flowing fast, they pinched the pipe, slowly releasing from time to time until they had finished feeding. Many of the mothers would also at times tear off a small piece of paper from the feeding chart and place it on the baby’s forehead. This they said they did to prevent the baby from getting hiccups which they believed could lead to reflux and potentially choking.

Throughout the first 24 h of admission the nurses on the ward concentrated on caring for the very sick babies, giving medication while the mothers took up the bedside monitoring and care of their babies often with little support from the nurses.

In the government funded hospital, in the days following the admission the mothers would settle into a routine. During the hours that followed the daily ward rounds by the medical team the mothers would sit by their baby’s incubator observing the baby, delivering primary care, such as diaper changing and 3 hourly feeding as well as some more specialized tasks such as the NG tube feeding, fixing oxygen masks in place for babies on oxygen and eye masks for babies on phototherapy, and observations of their babies while receiving phototherapy or on a respirator. The ways that most mothers learnt how to undertake these tasks was either through instruction from other mothers or by observing how the other mothers were completing similar task for their babies. Health care staff were rarely involved in providing support and instruction.

“I asked them whether they were taught how to toptail the baby, they all laughed and told me “where? One of them continued to narrate, *“here you are told to ask your neighbour, but it becomes difficult if you don’t have a nice neighbour willing to show you, so you just observe what the others are doing and practice”***(field notes-Government funded hospital)**.

*“No, no one taught me how to change him. It is just through looking at what others were doing, the way they are doing it you just do the same. We used to be told go and wash the babies, so now we don’t know where to start from, so we just look at how others are doing it and we also just do as the other one is doing”***[Government funded hospital_IDI_018]**.

This process of peer learning enhanced the friendships that developed amongst the mothers in the government funded hospital as they were together both in the wards and their sleeping areas, often even having to share a bed.

By contrast, in the faith-based hospital, care of the very sick babies and premature babies during the initial hospital admission hours was undertaken by nurses. Mothers played very little role in the care of their babies, with any access and activities being closely monitored by a nurse. Mothers were rarely present during, and did not participate in, the key tasks such as feeding, changing, and bathing of such babies.

*“Because like I said, when I came the first the day, I didn’t... I’m not the one who cleaned. She’s* (the nurse) *the one who cleaned the baby. She did it herself”***(Faith-based hospital_04)**.

**Fig. 2:**
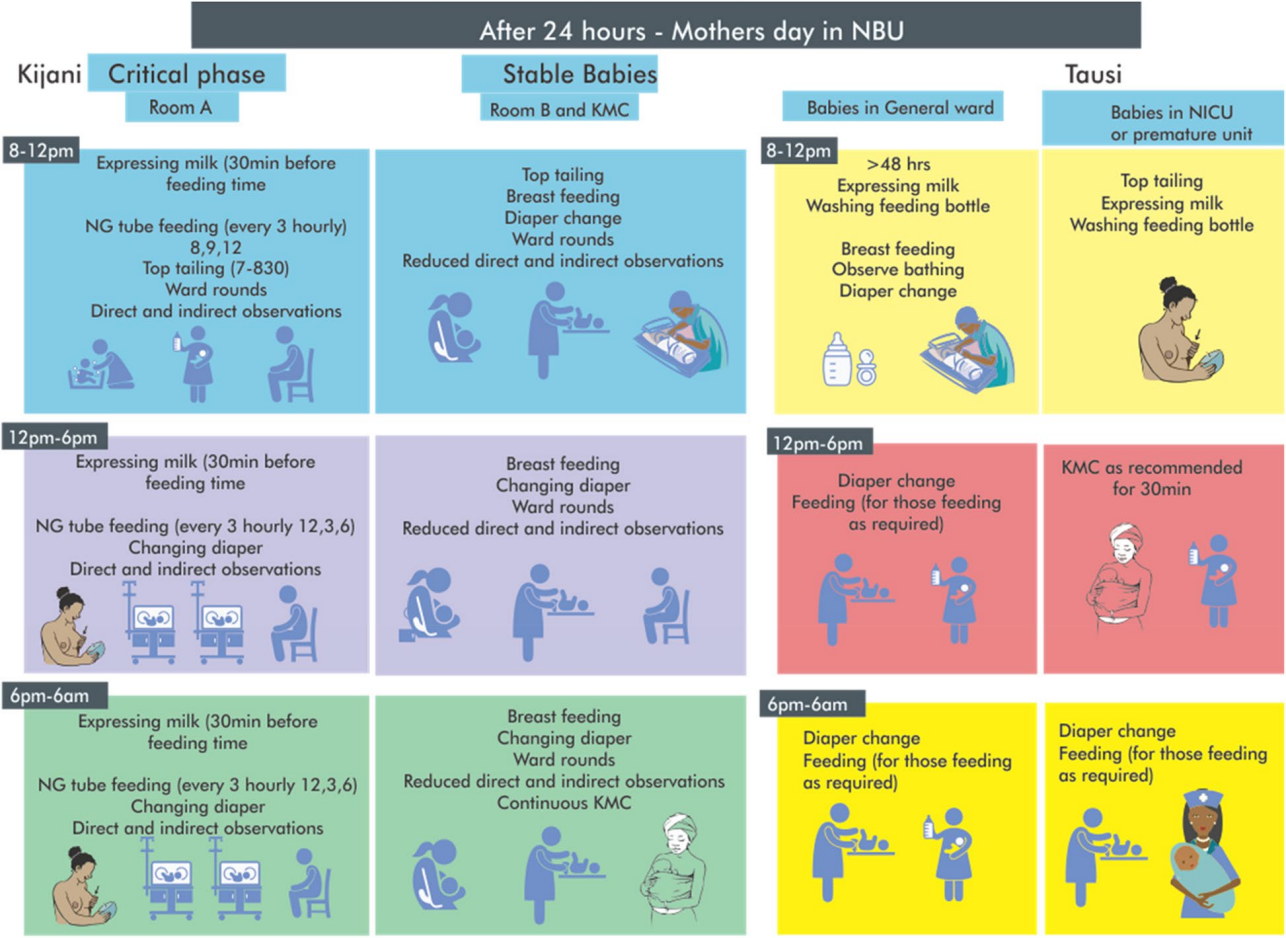
After 24 h

These mothers only started becoming actively involved in the care of their babies, taking on tasks such as feeding and bathing, several days after their baby’s admission (Table 1) and only after a baby had stabilised and been moved to the general ward. While the nurses acted as gatekeepers to the babies, the mothers did receive considerable support from them as the babies started to get better and the mothers became more involved in helping with care (Fig. 2). The process started with one-on-one learning sessions where mothers were progressively introduced to and taught how to perform tasks such as bathing by the nurses before eventually performing these tasks on their own.

*“The first time I washed the baby they asked me whether I’m comfortable washing the baby, if I have another baby, so washing the baby and I tried it, the nurse guided me, they were there and help me when I was unable to”.****(Faith-based hospital_05).***

Since the mothers were rarely continuously present in the faith-based hospital NBU and as many opted for voluntary discharge, the opportunities for getting to know the other mothers and develop peer support groups were limited. Consequently, the mothers in the faith-based hospital tended to rely on the nurses for support rather than each other.

A summary of the tasks that the mothers undertook in each hospital is provided in Figs. 1 and 2; Table 1.

### Mothers’ views on participation in care

In both hospitals, many of the mothers faced challenges in taking up the caring roles they were required to undertake, especially within the first few days. These challenges were related to their fears about the size of the baby as well as their ability to perform these tasks. In both hospitals, a key task expected of the mothers was expressing milk to feed their baby. But most of the mothers were not familiar with expressing milk and many struggled, feeling that they had insufficient milk for their babies. From our observations and even in their interviews, many of the mothers reported the challenges they faced with this task.

*“I even did not….they would insist that I express, but I did not express because I did not feel like…. I knew I had no milk, so I wondered what they expected me to express yet I did not have, I didn’t have!”***(Government funded hospital-IDI_07)**.

Neither hospital had adequate breastfeeding support interventions in place to address these challenges. Only on few occasions would a member of staff take time to sit and talk with the mothers and show them how to express or reassure them that their milk would come. In the government funded hospital mothers who were unable to express milk were sometimes scolded by the nurses or nutritionist without being given any suggestions as to how to do better. Support for these mothers was often provided by the other mothers in the NBU who offered advice to try and avoid stress or showed how to transfer the expressed milk into the NG feeding tube.

Expressing and feeding were particularly challenging for mothers who had delivered through C-section and those who had twins. The postnatal ward in the government funded hospital was on a different floor and some distance from the NBU which either meant walking long distances every 2 to 3 h for feeding or just spending most time sitting on a chair in the NBU with little opportunity for appropriate rest:

*“It was stressful, because my legs were still swollen and my stomach was still painful, when you are still breast feeding this one you are called to breast feed the other one, and when you walk you get tired, and at the same time you dont have milk, you just feel overwhlemed. You lack sleep, most of the time you are just sat on the chair. It was stressful”.***(Government funded hospital-IDI_010)**.

In the faith-based hospital mothers who were struggling with expressing milk had an alternative feeding option as they had access to the funds to purchase formula feeds and mixed feeding (formula and breast milk) was allowed and encouraged. Many of the mothers in the faith-based hospital were discharged home while their baby was still in the NBU and even though they were able to express and store their milk in a fridge either in the hospital or at home, formula feeding was often logistically easier. However, some of the doctors in the faith-based hospital were keen to ensure that the mothers did breastfeed. For some of the faith-based hospital mothers, there were elements of struggle with transitioning from bottled feeding to breastfeeding when the nurses had told them to start on formula milk (due to problems with producing milk) but the doctors then instructed that the baby should be receiving breastmilk:

*Breastfeeding? Breastfeeding, okay, first of all uhh, I think first of all my milk did not come immediately but it didn’t take long. So my baby I think was not getting enough milk and I remember first of all what they do*- [the nurse*], they tell you to buy the ‘Nan’* [formula milk] *just to introduce the baby so that so that the baby doesn’t stay hungry. Yeah, so you find that the baby becomes comfortable because with the bottle is faster and then now you want to change to this one that he has to use so much energy pulling. So I went and innocently told the doctor that that uhh the baby drinks formula milk but he doesn’t breastfeed. “No mun. This baby has to be, has to become very hungry. No formula milk, just breastfeed” the doctor responded. I think that is why I stayed for long hours because he was waking up every other time. So, yes, we tried breastfeeding the whole night. And I remember when I was cold* [breast feeding room was cold at night] *and I was thinking oh God, let them not call me because I can’t handle the baby. Let them not… I am just thinking I pray they don’t call me, they don’t call because I can’t carry the baby.***(Faith-based hospital_09)**.

Despite the struggles, the mothers across the two hospitals appreciated their involvement in care as it enabled them to bond with their babies. Mothers reported that getting involved in the daily care and being able to hold their babies and breastfeed enabled them to feel attached to their newborns:

*I have been involved in things like breastfeeding, at least you feel good when breastfeeding you feel good when you wash her, when you change diapers at least you start to bond. You hold her she keeps quiet, when she cries you hold her and she keeps quiet. I think this is the time now you become a mother*. **(Faith-based hospital_IDI_018)**.

However, in contrast to the mothers in the government funded hospital, many of the mothers in the faith-based hospital reported feeling powerless in the wards. As one of them said to DO in an informal conversation during the observations: ‘*I tend to come in only during visiting hours just to bring milk because even if I stay here, there is nothing much I can do anyway’*.

In the discharge interviews with mothers, we asked about how confident they felt about their ability to care for their babies post-discharge. Unlike the mothers in the government funded hospital, the mothers discharged from the faith-based hospital expressed their concerns about coping with the small baby at home without the support of the health care staff. This was less commonly reported by mothers from the government funded hospital who were present throughout the baby’s admission and much involved in the bedside care of their babies.

## Discussion

In this paper, we have described the contrasting roles that mothers play in caring for their sick newborns in two NBUs in Nairobi, Kenya. Geographically close, the two NBUs are embedded in strikingly different structural, economic and social environments and the roles that the mothers play in the care of their babies are directly shaped by these different environments. In general, the government funded hospital catered to women who were economically disadvantaged (compared to the women the faith-based hospital) and this disadvantage was echoed in the much inferior staffing ratios and the neglected and crowded environment. The understaffing in the government funded hospital contributed to the unplanned and informal high level of involvement of mothers in their children’s care, providing both primary care (breastfeeding, diaper changing, top-tailing) as well as some more specialised neonatal care such as NG tube feeding. It also impeded the level of support that nurses were able to offer mothers. Understaffing within the public sector has been documented as a bottleneck to service provision in LMICs [32]. A recent study by McKnight and colleagues [36], undertaken in Nairobi, found that nurses working within the government funded hospital setting are often overstretched, having to provide care in very challenging conditions. Working in overstretched and constrained environments can lead to the development of collective coping strategies and prioritization of care, emphasizing clinical care to the detriment of non-clinical aspects of care [36]. These findings echo those found in the studies in Iran where in understaffed NBUs the nurses delegated primary care to parents [33].

Having a child admitted to a hospital newborn unit is a stressful event for a parent, often mixed with fear, grief, and uncertainty [9, 45, 46]. Admission to a newborn unit disrupts the maternal-infant bonding and attachment process, an important factor in infant development and maternal wellbeing [47] as well a potential precursor to the consolidation of parenting skills and confidence, and future emotional, developmental and social milestones [7]. A recently published review of the literature on maternal-infant bonding reported that mothers who participated in immediate skin-to-skin contact and initiated breastfeeding within two hours following childbirth were more sensitive to the infant’s needs and the child seemed more content at one year; while poor interaction affects the child’s cognitive and socio-emotional development, physical health and personal relationships [7]. Paradoxically, given the widespread benefits to mothers and babies of maternal involvement in newborn care, it was the mothers in the government funded hospital who participated far more in the routine daily primary care of their babies. However, as was found in the Iranian study [48], this participation was frequently without adequate training and often with minimal support and supervision from health care staff in the NBU. Thus, although mothers in the government funded hospital gained experiential and lay knowledge that enabled them to develop expertise and confidence in the care of their babies [49], they had little choice in when and how they participated in care and the skills they gained were not always most appropriate. The lack of professional support and supervision by the health care staff had the potential for serious mismanagement of care with dangerous and possibly life-threatening consequences for the baby.

Staffing was not a challenge in the faith-based hospital and many mothers, for cost saving purposes, felt obliged to discharge themselves before their baby was ready to be discharged, leaving their babies under the care of nurses. This separation and limited initial participation of mothers in the care of babies may have contributed to stress and affected their well-being [11] as well as having potential consequences for their confidence in their ability to care for their baby post-discharge [40, 50].

In both hospitals the focus of the health care staff was on the immediate clinical needs of the babies with attention rarely given to the physical or psychological needs of the mother. This neglect of the mothers was visible both in the day-to-day interactions of the health care staff with the mothers and in the structural systems level in the physical separation of the mother and baby spaces. The day-to-day interactions between the staff and the mothers focused on the needs of the baby with little or no support for the mothers in their struggles to express milk or breast feed, and a lack of attention to their social and emotional well-being. At the structural systems level in the faith-based hospital no financial support was provided to help the mothers stay in the hospital near to their baby and in the government funded hospital the accommodation for mothers was on a different floor and distant from the NBU, constraining their ability to both get adequate rest and participate effectively in the care of their babies. Ward lay out and consideration of the mother-infant dyad are significant concerns influencing the ability of mothers to effectively participate in the care of their hospitalised sick newborns [51, 52].

In the recent iKMC trial the participating hospitals modified their existing neonatal intensive care units (NICUs), or purpose-built new units, to create Mother-NICUs (MNICUs) where each mother-baby dyad was provided with a bed to allow for the practice of iKMC. In addition, the provision of care within these units was provided by teams of obstetricians and neonatologist, addressing the needs of both mother and baby [23]. This strategy not only facilitates the implementation of iKMC but also provides the structure through which a more holistic approach to the delivery of neonatal care could be implemented. Taking into account the needs of the mother is the first step towards family centred care and the concepts of parental involvement and team work [41, 44–46].

LMIC settings, including Kenya struggle with numerous health system challenges such as understaffing and inadequate infrastructure which impact the implementation and uptake of interventions such as FCC. However, we need to begin to critically think about contextually and culturally appropriate interventions that can build on the opportunities that exist to improve care within the NBU in line with the WHO standards in such highly resource constrained settings. We argue that health providers and mothers need to work together and develop a strategic focus on the clinical, emotional and social needs of mother-baby dyad. The iKMC approach clearly has potential but requires significant system changes and resourcing. As a first step, the skills of nurses to better identify and support the needs of mothers and their families need to be developed and implemented. In particular, mother infant bonding within the first 24 h should be encouraged across all hospitals and strategies to better equip mothers and supervise their participation in care need to be established. Central to any approach needs to be consideration of strategies to address the chronic understaffing seen in many government funded hospitals in LMICs.

### Strengths and limitations

The key strength of this study was in the ability to triangulate findings across two different hospital contexts across 2 sectors. Non-participant observations provided a more nuanced understanding of the context of newborn care in an inpatient setting. This study also begins to shed some light on how hospital norms and contextual factors shape the support mothers receive and influence their participation in care which has implications for FCC. However, this study only focussed on understanding mothers’ experiences and did not document the voices of the nurses. The data reported in this manuscript was collected between 2017 and 2018. However, ongoing work within public hospitals in Kenya suggests that staffing and the structural contexts of care have not changed considerably with the most government funded facilities still experiencing the reported bottlenecks.

## Conclusion

This qualitative study of care provision in the neonatal units of a government funded and a faith-based hospital in Kenya points to stark differences between the care environment available for women of different socio-economic classes. Our findings suggest understaffing, and less formal support for mothers in the government funded sector, which necessitated the development of stronger peer support bonds between mothers than was observed in the faith-based hospital. While higher levels of involvement and participation in bedside care can be beneficial for mothers, there is a need for approaches to improving care within neonatal units that take account of the perspectives of mothers and staff, as well as advocacy for structural changes to support respectful care and reduce inequities in neonatal care. Focusing on technological improvements risks ignoring structural inequities and further embedding a biomedical paradigm that ignores the relationships and needs of mothers, families, and staff. Advances in medical care are necessary but not enough to address the disrespect and persistent structural inequities affecting access to and quality of care. A first step is recognising the importance of the mother/infant dyad in the NBU; understanding who is providing ‘care’ in the NBU and drawing on the experiences of mothers, families, and staff to develop interventions that address their needs. This involves treating mothers with respect through a recognition of their role as key partners in care provision; leveraging their agency and developing their cultural competence in providing care for their sick newborns.

## Supplementary Information

**Additional file 1.**

## Data Availability

The datasets generated and/or analysed during the current study are not publicly available due the small study sample which makes the participants easily identifiable but are available from the corresponding author on reasonable request.

## Abbreviations

FCC: Family Centred Care
HIC: High Income Contexts
iKMC: immediate Kangaroo Mother Care
LMICs: Low and middle-income countries
NG: Nasogastric
NBUs: Newborn units
WHO: World Health Organisation
