# Supplementary material for: Mothers’ involvement in providing care for their hospitalised sick newborns in Kenya: a focused ethnographic account
Source: BMC Pregnancy Childbirth. 2023 May 26;23:389. doi: 10.1186/s12884-023-05686-3 (PMC10214722; doi:10.1186/s12884-023-05686-3)
Supplement: Supplementary file 1 — Additional file 1. [file 12884_2023_5686_MOESM1_ESM.docx]

**ANNEX 1**

Socio-demographic representation of the mothers in the two study hospitals-Discharge interview data

| Variable | Classification | Government funded hospital (n=20) | Faith based hospital (n=20) |
| --- | --- | --- | --- |
| Age (Years) | Below 20  20-25  26-30  31-35  >35 | 2  6  8  1  3 | 0  6  7  4  3 |
| Education | None  Primary  Secondary  Tertiary | 1  7  10  2 | 0  0  1  19 |
| Occupation | Unemployed  Casual*  Semi-skilled**  Employed***  Other | 8  4  7  1  0 | 3  0  0  17  0 |
| Marital status | Single  Married | 6  14 | 2  18 |

- *Casual* Lack of any tertiary skills training*
- *Semi-skilled** Possess minimal skills training*
- *Employed***Posses extensive skills training and are mostly informal employment*
